# Use of Natural Family Planning (NFP) and Its Effect on Couple Relationships and Sexual Satisfaction: A Multi-Country Survey of NFP Users from US and Europe

**DOI:** 10.3389/fpubh.2017.00042

**Published:** 2017-03-13

**Authors:** Matthias Unseld, Elisabeth Rötzer, Roman Weigl, Eva K. Masel, Michael D. Manhart

**Affiliations:** ^1^Department of Internal Medicine I, Medical University of Vienna, Vienna, Austria; ^2^Institute of Natural Conception Regulation, Vienna, Austria; ^3^Couple to Couple League, Cincinnati, OH, USA

**Keywords:** natural family planning, multi-country study, sympto-thermal method, relationship, sexual satisfaction

## Abstract

**Purpose:**

Birth control is a persistent global health concern. Natural family planning (NFP) comprises methods to achieve or avoid pregnancy independent of mechanical or pharmacological intervention. The sympto-thermal method (STM) of NFP employs daily observation of cervical fluids and measurement of basal body temperature. This multi-country study was undertaken to describe the characteristics of STM users, understand their perceptions of NFP, and its perceived impact on relationships.

**Methods and results:**

Questionnaires for women and men were developed in German and translated to English, Polish, Italian, Czech, and Slovak by native speakers. A total of 2,560 respondents completed the online questionnaire (37.4% response). Participants were married (89%) and well educated, and their self-perceived financial status was described as “good” or “very good” by 65% of the respondents. Forty-seven percent had previously used contraceptives. Ninety-five percent of women and 55% of men said using NFP has helped them to know their body better. Large majorities of men (74%) and women (64%) felt NFP helped to improve their relationship while <10% felt use of NFP had harmed their relationship. Most women (53%) and men (63%) felt using NFP improved their sex life while 32% of women and 24% of men felt it was unchanged from before they used NFP. Seventy-five percent of women and 73% of men said they are either “satisfied” or “very satisfied” with their frequency of sexual intercourse.

**Conclusion:**

This survey demonstrates STM of NFP is a well-accepted approach to family planning across several Western cultures. It is consistently viewed as being beneficial to couples’ self-knowledge, their relationship, and satisfaction with frequency of sexual intercourse.

## Introduction

Fertility awareness-based methods (FABM) of family planning use biologic markers to identify fertile and infertile days of a woman’s reproductive cycle. Sexual intercourse is avoided or contraception is used on fertile days to prevent pregnancy. Natural family planning (NFP) is a type of FABM that employs abstinence during the fertile window if being used to postpone pregnancy (1). Different modern methods of NFP are available for family planning (2, 3), and several have a good level of medical evidence for their effectiveness in avoiding pregnancy (4).

Natural family planning offers couples the opportunity to approach fertility as a normal biological process and, by synchronizing their sexual behavior with the normal periodicity of fertility, can plan their families while respecting possible cultural and/or religious beliefs they may have.

Despite this, many question NFP as a viable family planning method due to its dependence on user compliance and self-control during the fertile period (5). Previous surveys of users of NFP indicate the daily habit and periodic abstinence in general is not burdensome, and many claim it has benefits to their overall relationship (6–8). Participants in these studies used cervical mucus or temperature only methods of NFP.

The sympto-thermal method (STM) of NFP combines cervical mucus observation with basal body temperature (BBT) recordings to provide a “cross checking” system that has been shown in high quality clinical studies to have unintended pregnancy rates of <1% with perfect use and 2–8% with typical use (9–11).

This prospective multi-country study was undertaken to describe the characteristics of STM users, understand their perceptions of NFP, and its perceived impact on relationships. A single survey integrating both male and female questionnaires was distributed in the US and seven European countries using the membership lists of two large NFP organizations providing the opportunity to explore similarities and differences among NFP users across cultural landscapes.

## Materials and Methods

The study was performed following the institutional Good Scientific Practice standards of the Medical University of Vienna and has been conducted according to the principles expressed in the Declaration of Helsinki. The Internet-based survey collected responses anonymously and so was declared exempt from requiring informed consent by the Ethics Committee of the Medical University of Vienna.

The participants for this international descriptive study were NFP users from two major NFP organizations in the US [Couple to Couple League (CCL)] and Europe (Institut für Natürliche Empfängnisregelung, INER).

Two questionnaires, one for women and one for men, were developed for use as an online instrument on NFP topics. The questionnaire was translated from German to English, Polish, Italian, Czech, and Slovak by native speakers prior to distribution in respective countries. The survey included questions on socioeconomic, health, gynecological, NFP, and sexual topics. The survey took place between February 2015 and April 2015. Potential respondents were invited to participate *via* an electronic invitation that described the purpose of the survey, assured anonymity, and contained links to both the male and female questionnaire. The survey was hosted on an online polling service that provides a unique link for each participant that cannot be traced to them (Q-set, Nittenau, Germany http://www.q-set.at). This way an individual remains anonymous, yet all of their specific responses remain aligned in the dataset, allowing for detailed analyses.

To minimize selection bias in recruitment, the invitation was emailed to all addresses on the current email lists of CCL and INER. Of the 6,827 opened invitations (3,750 in US and 3,077 in Europe), 2,560 completed the questionnaire; an overall response rate of 37.4% (32.4% in US and 43.7% in Europe).

The study population was characterized by descriptive statistics, Kruskal–Wallis test, and was tested pairwise by Student’s *t*-test. Responses by country were analyzed by SAS procedures FREQ including Nopercent, nocum, binominal, and exact Fisher/MC (SAS Enterprise Guide 6.1 based on SAS 9.4, SAS Institute Inc., Cary, NC, USA) with *p* < 0.05 as significance level.

## Results

### NFP Survey Participants

A total of 2,560 participants completed the questionnaire; 77% (*n* = 1,971) were females, and 23% (*n* = 589) were males. Respondents from the US constituted 47% of the total while 30% took part from German-speaking countries (Austria, Germany, and Switzerland) in Europe with the remaining from Slovakia, Poland, Czech Republic, and Italy (Table 1).

**Table 1:** **Distribution of respondents by country and sex**.

Country	US	DE, AT, and CH	SK	PL	CZ	IT
Total, *n* (%)	1,214 (47)	781 (30)	169 (7)	176 (7)	153 (6)	67 (3)
Female, *n* (%)	965 (79)	575 (74)	138 (97)	136 (77)	107 (70)	50 (75)
Male, *n* (%)	249 (21)	206 (26)	31 (3)	40 (23)	46 (30)	17 (25)

*US, Untied States; DE, Germany; AT, Austria; CH, Switzerland; PL, Poland; CZ, Czechoslovakia; IT, Italy. DE, AT, and CH were only provided German questionnaires so this cluster represents German-speaking European respondents*.

Table 2 describes various characteristics of the respondent pool. As expected, over 80% of the respondents were of reproductive age; 52% were between 31 and 50 years old while 36% were between 18 and 30 years. Most of the participants were married (89%) and well educated; 73% holding a university degree. Self-perceived financial status was described as “good” (46%) or “very good” (19%) by most of the respondents.

**Table 2:** **Characteristics of survey respondents**.

Age	<18	18–30	31–50	>50	
*n* (%)	2 (<1)	912 (36)	1,329 (52)	315 (12)	
Education	Compulsory	Vocational	Secondary	University	
*n* (%)	47 (2)	229 (9)	404 (16)	1,878 (73)	
Family status	Single	Cohabiting	Married	Widowed	Divorced
*n* (%)	130 (5)	119 (5)	2,286 (89)	7 (<1)	18 (<1)
Financial situation	Very good	Good	Enough	Not enough	
*n* (%)	475 (19)	1,187 (46)	826 (32)	69 (3)	
Natural family planning (NFP) type used	Sympto-thermal method	Temp	Cervical mucus	Other	
*n* (%)	1,956 (80)	95 (4)	256 (10)	117 (5)	
How learned NFP^a^	Book	Course	Friends	Other	
*n* (%)	1,382 (54)	1,864 (73)	617 (24)	365 (14)	
NFP org. member	No	INER	CCL	Other	
*n* (%)	1,354 (55)	383 (15)	663 (27)	79 (3)	
Number of children	0	1–2	3–4	≥5	
*n* (%)	538 (27)	653 (33)	541 (28)	227 (12)	

*^a^Multiple responses possible*.

User characteristics were generally consistent among respondents from different countries with few noted differences. Compared to non-US respondents, significantly more US respondents reported a university degree (73 vs 91%, respectively, *p* < 0.001) and significantly more rated their financial situation as “very good” (19 vs 24%, respectively, *p* < 0.0001). German-speaking respondents had a significantly higher proportion of persons over 50 years of age (18 vs 10%, *p* < 0.001) and significantly more cohabiting households (11 vs 2%, *p* < 0.001) when compared to non-German-speaking respondents.

The average duration of NFP use was 8.5 years (± 8.0 years) with a range of 0–35 years. Ninety-five percent (2,406 out of 2,525) of all responders are current or former NFP users. Eight in 10 respondents are practicing or practiced the STM, the method taught by both CCL and INER. Most of the respondents learned NFP through attending a course (73%) and/or reading an NFP book (54%; multiple answers possible for this question). Despite the study pool being generated from CCL and INER mailing lists, over half of respondents indicated they are currently not a member of any NFP organization. Membership to CCL was reported by 27%, and membership to INER was reported by 15%.

### NFP and Family Size

Respondents most commonly had either no children, one to two children, or three to four children (Table 2). Large families, defined here as five or more children, were uncommon, and the frequency varied by country. Significantly more US respondents had large families (16%, *p* < 0.001) compared to all non-US respondents, potentially reflecting their significantly higher self-perceived financial status. Only 2% of Italian respondents reported large families while the remaining countries had between 4 and 8% of respondents with large families.

At the time of this study, 80% of women were using NFP to avoid/postpone pregnancy. Among those currently trying to conceive, 68% (258 out of 379) have been trying for less than a year. More than half of all female respondents reported they became pregnant in 6 months or less when they were trying to conceive and fewer than 9% indicated they had to try for longer than a year to conceive. It is likely this cohort of women was aware when they were fertile in their cycles and thus conceived relatively soon after starting to try; underscoring the value of fertility awareness when trying to conceive.

### Contraceptive Use

Almost half of respondents claimed to have used contraceptives previously, suggesting that many users discovered NFP only after trying other methods of family planning. Among men, 48% (260 out of 549) claimed previous use, 45% (*n* = 249) never used, and 7% (*n* = 40) stated they currently use a contraceptive. Women were asked to differentiate between hormonal and barrier contraceptive use. Previous use of barriers was reported by 41% (773 out of 1,893), never use by 49% (*n* = 938), and current use by 10% (*n* = 182). Previous use of hormonal contraceptives was reported by 44% (815 out of 1,959), never use by 56% (*n* = 1,096), and current use by <1% (*n* = 15). Of those who previously used hormonal contraceptives, most used for 1–3 years or 4–10 years. Previous use of barrier contraceptives was more commonly for shorter durations.

### NFP and Relationship Dynamics

Table 3 provides responses to questions related to the effect of NFP on relationship dynamics. Ninety-five percent of women and 55% of men said that using NFP has helped them to know their body better. Among those who had used NFP for <1 year, 92% (*n* = 297) felt NFP helped them to know their body better while only 2% (*n* = 7) disagreed with this.

**Table 3:** **Natural family planning (NFP) effects on relationship**.

**Since using NFP I have gotten to know my body better**.				
	Yes, *n* (%)	Same, *n* (%)	No, *n* (%)	
Women	1,766 (95)	80 (4)	17 (1)	
Men	314 (55)	198 (35)	59 (10)	
**NFP has helped to develop our relationship.^a^**				
	Yes, *n* (%)	Same, *n* (%)	No, *n* (%)	
Women	1,145 (65)	469 (26)	161 (9)	
Men	409 (74)	97 (17)	49 (9)	
**How important to you is your partner’s interest in NFP?**^a^				
	Very important, *n* (%)	Important, *n* (%)	Not very important, *n* (%)	Not at all important, *n* (%)
Women	994 (74)	268 (20)	27 (2)	49 (4)
Men	275 (81)	50 (15)	9 (2)	6 (2)
**My knowledge of NFP helps (or has helped) me to explain sexuality to my children.^b^**				
	Yes, *n* (%)	No, *n* (%)	NFP too late to help, *n* (%)	
Women	693 (85)	107 (13)	10 (1)	
Men	251 (82)	53 (17)	1 (<1)	

*^a^Among those with a spouse/partner*.

*^b^Among those with children*.

Large majorities of both men (74%) and women (65%) stated NFP had helped to improve their relationship or it had made little difference (17% of men and 26% of women). Importantly, less than 10% of men and women felt their use of NFP had harmed their relationship. The potential influence of educational level on the relationship was explored by comparing the responses of men and women with a university degree to those with less than a university degree; in both men and women responses to this question were similar regardless of educational level (data not shown). Both men and women recognized the involvement and commitment of their partner is important for use of NFP; 94% of women and 96% of men felt their partner’s interest is either “very important” or “important.” Among those with children, 85% of women and 82% of men indicated the knowledge gained by using NFP helped them to explain sexuality to their children.

### NFP and Sexuality

Table 4 provides responses to questions related to the effect of NFP on couples’ intimate life. Most women (69%) and men (72%) felt NFP has helped them speak about sexuality to their partner while less than 8% felt it did not. More than 80% of both men and women felt NFP improved their knowledge and understanding of sexuality. The potential influence of educational level on knowledge and understanding of sexuality was explored by comparing the responses of men and women with a university degree to those with less than a university degree; responses to this question in both sexes were similar regardless of educational level (data not shown).

**Table 4:** **Effect of natural family planning (NFP) on sex life**.

**NFP helps (or helped) me speak about sexuality in my relationship**.				
	Yes, *n* (%)	Same, *n* (%)	No, *n* (%)	
Women	1,230 (66)	445 (24)	111 (6)	
Men	402 (71)	115 (20)	41 (7)	
**Since using NFP, I have found my sex life more joyful and enjoyable.^a^**				
	Yes, *n* (%)	Same, *n* (%)	No, *n* (%)	
Women	915 (62)	550 (37)	14 (1)	
Men	348 (63)	138 (25)	63 (11)	
**NFP has improved my knowledge and understanding of sexuality**.				
	Yes, *n* (%)	Same, *n* (%)	No, *n* (%)	
Women	1,501 (81)	304 (16)	49 (3)	
Men	475 (83)	81 (14)	14 (2)	
**In recent months, how often have you had sexual intercourse?**				
	Never, *n* (%)	≤1×, *n* (%)	1–3×, *n* (%)	≥4×, *n* (%)
Women	158 (8)	193 (10)	618 (32)	958 (50)
Men	39 (7)	83 (14)	179 (30)	286 (49)
**Are you satisfied with the frequency of sexual intercourse?**				
	Very satisfied, *n* (%)	Satisfied, *n* (%)	Unsatisfied, *n* (%)	Very unsatisfied, *n* (%)
Women	445 (23)	925 (48)	387 (20)	59 (3)
Men	132 (23)	291 (50)	129 (22)	25 (5)

*^a^Among those with spouse/partner*.

Among those in a relationship, 62% of women and 63% of men said that NFP improved their sex life while 37% of women and 25% of men felt it was unchanged from before they used NFP. Approximately 1% of women and 11% of men felt use of NFP had harmed their sex life.

Reported frequency of sexual intercourse was consistent between men and women; nearly half of respondents had sex four or more times per month. Not surprisingly frequency of sexual intercourse declined with age; 60% (493 out of 817) of those 18–30 years old, 49% (633 out of 1,274) of those 31–50 years old, and 39% (116 out of 299) of those over 50 years old reported intercourse four or more times per month. Seventy-five percent of women and 73% of men said they were either “satisfied” or “very satisfied” with their frequency of sexual intercourse, slightly over 20% were unsatisfied, and less than 5% were very unsatisfied. Interestingly, men with less than a university degree were significantly more likely to report being “very satisfied” or “satisfied” with their frequency of sexual intercourse compared to men with a university degree (81 vs 70%, respectively, *p* = 0.011) whereas women with differing levels of education showed no difference in satisfaction levels (77% vs 75%, *p* = 0.416).

## Discussion

This large multi-country survey provides important insights on the experience of using NFP as a method of family planning. In this study, majorities of men (55%) and women (95%) felt using NFP has helped them to better understand their bodies. Nearly two-thirds of men and three-quarters of women felt that the use of NFP improved their relationship while fewer than 10% felt it had not. For those with children of an appropriate age, over 80% felt using NFP helps explain sexuality to their children.

In general, a large majority of couples feel using NFP has helped them speak about their sexuality with their partner, improved their sex life, and improved the knowledge and understanding of their sexuality. Although NFP does require periodic abstinence if postponing pregnancy, three-quarters of men and women are satisfied or very satisfied with their frequency of sexual intercourse.

Our results are consistent with and expand upon similar previous surveys of NFP users. VandeVusse (8) found among 334 couples using mucus-only methods that 74% felt their use of NFP resulted in stronger bonds, better communication in their relationship and further improved knowledge on sexual issues. Fehring (7) has recently reported on this same cohort using the questions from Marshal and Rowe’s (6) survey of BBT method users. He found that overall satisfaction was 20% points higher among users of the modern NFP method compared to BBT users, and 80% of men and 85% of women felt NFP helped their marriage.

Oddens (12) surveyed German female medical students and found satisfaction with NFP was significantly lower (43%) than users of hormonal contraceptives (68%), but sexual pleasure was significantly higher for NFP users. This study is limited in that the specific type of NFP used is unknown. Other general surveys indicate many who claim to be using a natural method are in fact practicing some self-styled variation of the rhythm method NFP (13, 14).

The WHO five-country clinical trial of the Ovulation method (15) found user satisfaction with the frequency of sexual intercourse lower in those living in developed countries compared to those in developing countries; 63–65 vs 96–97%, respectively. Our results from well-educated persons living in Western Europe and the US using STM fall between these (73% of men and 75% of women).

The study reported here builds upon earlier studies in several ways. First, the size of the respondent pool is large; our survey included over 2,500 individuals compared to less than 500 in previous work. Second, more than 80% of respondents were STM users—a well-researched method for effectiveness but one that heretofore has been largely unexplored for the lived experience of real-world users. Finally, our survey captured responses from seven different countries allowing the conclusions to be more generalizable than previous single country studies.

This study is limited in that the profile of NFP users here represents a generally married, well-educated, and financially secure cohort. Thus, applicability of these results to less well-educated populations, persons who do not feel as financially secure, or are not in long-term relationships is limited. It is also by necessity a convenience sample; participants are limited to those who were on the membership lists of two large NFP organizations and who chose to respond to the invitation to complete the survey. The significantly larger female respondent pool reflects the voluntary nature of the sample and is consistent with other unpublished Internet surveys distributed to CCL member households (surveys seeking both spouses’ responses are 70–80% female respondents).

The WHO Department of Reproductive Health and Research envisions a world where all people can attain the highest possible level of sexual and reproductive health and where everyone has access to sexual and reproductive health information (16). The results of this and earlier studies suggests that for at least an important minority, modern NFP methods more broadly promoted and adopted would help move toward this vision. In a representative cohort of reproductive-aged US women, despite universal sex education in school, widespread access to contraceptives, and contraceptives legislated as an essential preventative service, approximately 40% of reproductive age women are unfamiliar with their ovulatory cycle (17). In contrast, NFP users are well aware of their ovulatory cycle, and this study indicates that this knowledge helps them as parents educate their own children; multiplying the impact of the investment to promote and support adoption of NFP in communities.

In conclusion, the results of this study underscore the benefit of NFP promoted and offered as a viable family planning option. Since NFP education can be delivered by non-medically trained educators and ongoing costs are virtually non-existent, the public health burden is minimal. These results demonstrate that at least in developed countries, NFP is well accepted by users and is perceived to have benefits that promote and support stable lifelong family relationships.

## Author Contributions

Conceived and designed the survey: MU and ER. Acquisition, analysis, and interpretation the data: RW, EM, MU, MM, and ER. Wrote the paper: MU and MM.

## Conflict of Interest Statement

The authors declare that the research was conducted in the absence of any commercial or financial relationships that could be construed as a potential conflict of interest. MM is currently an unpaid employee of the Couple to Couple League (MM was a paid employee and Executive Director for CCL until June 2016 and now serves in a scientific consulting role). ER is president of the Institute of Natural Conception Regulation, which is an unpaid employee position. The remaining authors are not associated with organizations that could be perceived as influencing the work and report no other potential conflicts.
